# Discovering a binary CTCF code with a little help from BORIS

**DOI:** 10.1080/19491034.2017.1394536

**Published:** 2017-12-05

**Authors:** Victor V. Lobanenkov, Gabriel E. Zentner

**Affiliations:** a Molecular Pathology Section, Laboratory of Immunogenetics, National Institute of Allergy and Infectious Diseases, National Institutes of Health, 5601 Fishers Ln, Rockville, MD, USA; b Department of Biology, Indiana University, 915 E 3^rd^ St, Bloomington, IN 47405, USA

**Keywords:** CTCF, CTCFL, BORIS, 1xCTS, 2xCTS, chromatin, ChIP-seq

## Abstract

CCCTC-binding factor (CTCF) is a conserved, essential regulator of chromatin architecture containing a unique array of 11 zinc fingers (ZFs). Gene duplication and sequence divergence during early amniote evolution generated the CTCF paralog Brother Of the Regulator of Imprinted Sites (BORIS), which has a DNA binding specificity identical to that of CTCF but divergent N- and C-termini. While healthy somatic tissues express only CTCF, CTCF and BORIS are normally co-expressed in meiotic and post-meiotic germ cells, and aberrant activation of BORIS occurs in tumors and some cancer cell lines. This has led to a model in which CTCF and BORIS compete for binding to some but not all genomic target sites; however, regulation of CTCF and BORIS genomic co-occupancy is not well understood. We recently addressed this issue, finding evidence for two major classes of CTCF target sequences, some of which contain single CTCF target sites (1xCTSes) and others containing two adjacent CTCF motifs (2xCTSes). The functional and chromatin structural features of 2xCTSes are distinct from those of 1xCTS-containing regions bound by a CTCF monomer. We suggest that these previously overlooked classes of CTCF binding regions may have different roles in regulating diverse chromatin-based phenomena, and may impact our understanding of heritable epigenetic regulation in cancer cells and normal germ cells.

## CTCF and BORIS

CTCF is an essential, ubiquitously expressed DNA-binding factor conserved from *Drosophila* to human.^1^ CTCF is a master regulator of chromatin architecture and participates in transcriptional activation and repression,^2–5^ imprinting,^6,7^ chromatin insulation,^8,9^ formation of higher-order chromatin structures,^10–12^ and X-chromosome inactivation^13^ and inactivation escape,^14^ among many other chromatin-based phenomena. In addition to CTCF, vertebrates express a paralogous gene termed Brother Of the Regulator of Imprinted Sites (BORIS, also known as CTCFL).^15^ BORIS arose from a gene duplication early in the evolution of amniotes^16^ and subsequent divergence from the ancestral CTCF sequence during vertebrate evolution. The regulation of CTCF and BORIS in humans and in other placental mammals has diverged to such an extent that they are co-expressed only during gametogenesis, while all normal embryonic and adult somatic tissues are BORIS-negative due to methylation of the *BORIS* promoters^17^ and express only CTCF. The *CTCF* gene evolved in chromosomal contexts homologous to the human 16q22 region, recognized for recurrent LOH in tumors,^18^ while its paralog *BORIS* has co-evolved in different chromosomal bands homologous to 20q13, known for amplification during immortalization in culture and cancerous transformation *in vivo*.^19^ In line with its germline-restricted expression, BORIS-null male mice have notable infertility due to meiotic defects.^20,21^ BORIS is also activated in a variety of human cancers and so is classified as a cancer-testis antigen (CTA)^22^ shown to be suitable for immunotherapy in animal models.^23^

**Figure 1.**
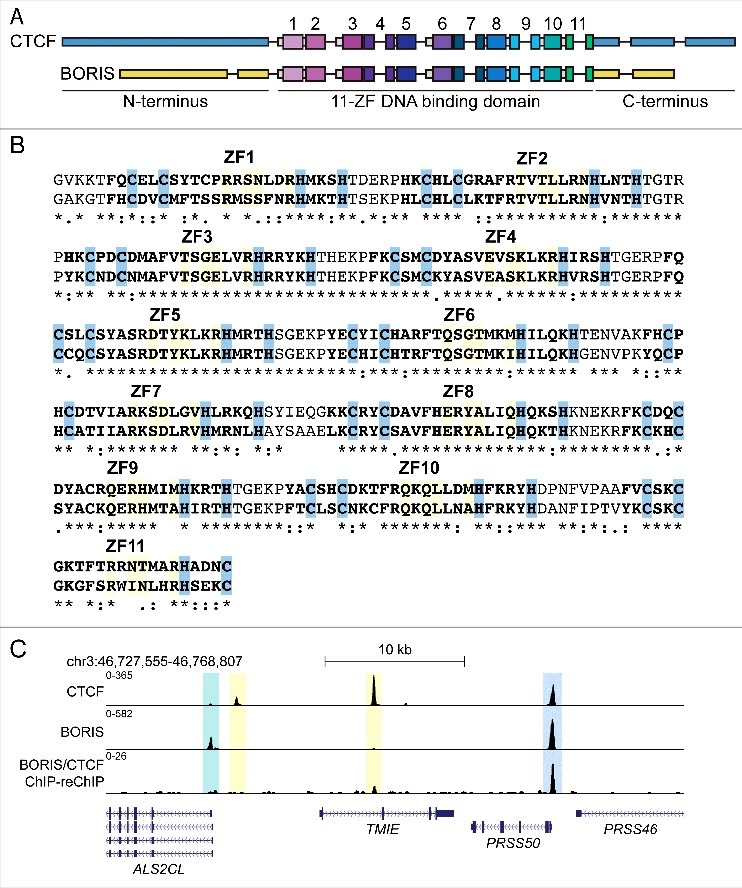
The DNA binding domains of CTCF and BORIS are highly conserved and bind to shared and distinct sites in the human genome. (A) Schematic representations of the genomic architecture of the *CTCF* and *BORIS* genes. C- and N-terminal exons are represented by thin blue (CTCF) or yellow (BORIS) lines. Conserved ZF-encoding exons are represented by thick colored boxes, with exons corresponding to each ZF colored differently and the exon(s) numbers according to which ZF they encode. ZFs 4, 7, 9, and 11 are split between two exons; in these cases, the ZF number is written above the intervening intron. Portions of exons corresponding to ZF linkers are represented by thin grey boxes. Introns are represented by black lines. (B) Alignment of the 11-ZF DNA binding domains of CTCF and BORIS. Zinc fingers are in bold and numbered. Zinc-coordinating residues are highlighted in blue and DNA-contacting residues are highlighted in gold. The asterisk (*) indicates amino acid conservation, the colon (:) represents a strongly conservative amino acid substitution, and the period (.) represents a weakly conservative amino acid substitution. The alignment was generated with Clustal Omega. (C) Human K562 cell ChIP-seq and ChIP-reChIP-seq data demonstrating the presence of two major classes of CTCF and BORIS binding regions. A robust CTCF&BORIS-bound 2xCTS element at the *PRSS50* (*TSP50*) gene promoter has been characterized in detail in an earlier study^24^ and verified by analysis of DNase I footprinting data^26^ as well as ChIP-reChIP-seq (BORIS ChIP with antibodies verified for lack of CTCF crossreactivity followed by CTCF reChIP-seq) is highlighted in blue. Two CTCF-only peaks are highlighted in yellow, and a BORIS-only peak is highlighted in green.

The central 11-ZF DNA binding domains of CTCF and BORIS are highly conserved in terms of genomic architecture and amino acid sequence^15^ (Fig. 1A-B) and have identical DNA binding specificities. The existence of at least two cellular settings (male germ cells and cancer cells) in which CTCF and BORIS are co-expressed raises the question of whether they associate cooperatively or competitively with a finite number of recognition sequences in the genome. A favored model is that of zero-sum competition, in which one protein completely replaces the other at a given target site. This model has been proposed to be supported by ChIP-seq analysis of CTCF and BORIS, showing single peaks of both paralogous 11-ZF proteins overlapping at thousands of genomic loci.^21^ However, this model does not account for a number of binding regions that contain two closely spaced CTCF motifs simultaneously bound in EMSA experiments by not one but two 11 ZF DBDs of both paralogs,^24,25^ which apparently cannot be resolved by ChIP-seq experiments with CTCF-specific antibodies alone.

## Genome-wide characterization of CTCF and BORIS binding

To understand the global patterns of selective DNA occupancy by co-expressed CTCF and BORIS, we performed comprehensive ChIP-seq and ChIP-reChIP-seq analyses of both proteins in several human somatic cancer cell lines as well as mouse post-meiotic round spermatids.^26^ This revealed tens of thousands of specific peaks for both proteins in the BORIS-positive K562, OVCAR8, and Delta47 cancer cell lines. CTCF but not BORIS peaks were also observed in normal human dermal fibroblasts, used as a positive control for CTCF ChIP-seq and a negative control for BORIS ChIP-seq. Strikingly, 29–38% of all detected CTCF peaks overlapped with BORIS peaks. ChIP-reChIP-seq analysis in myeloid K562 and lymphoid Delta47 cells confirmed simultaneous occupancy of CTCF and BORIS at these overlapping binding regions. A similar result was observed in purified post-meiotic mouse round spermatids, where 25% of CTCF peak regions were also occupied by BORIS. Regions co-bound by CTCF and BORIS contained at least two closely-spaced, robust CTCF binding motifs, while CTCF-only peaks contained one or no match to an established CTCF motif.^27^ Due to their motif content, we termed CTCF-bound ChIP-seq peak regions also displaying BORIS recruitment as 2xCTSes and those bound by CTCF alone 1xCTSes. We also observed a number of 2xCTSes bound by homodimeric BORIS, though these were fewer in number than the 2xCTS elements co-bound by CTCF and BORIS together in BORIS-positive cells. A representative genome browser view of ChIP-seq and ChIP-reChIP-seq data from K562 cells showing distinct CTCF and BORIS binding features of 1xCTS and 2xCTS elements is displayed in Fig. 1C.

**Figure 2.**
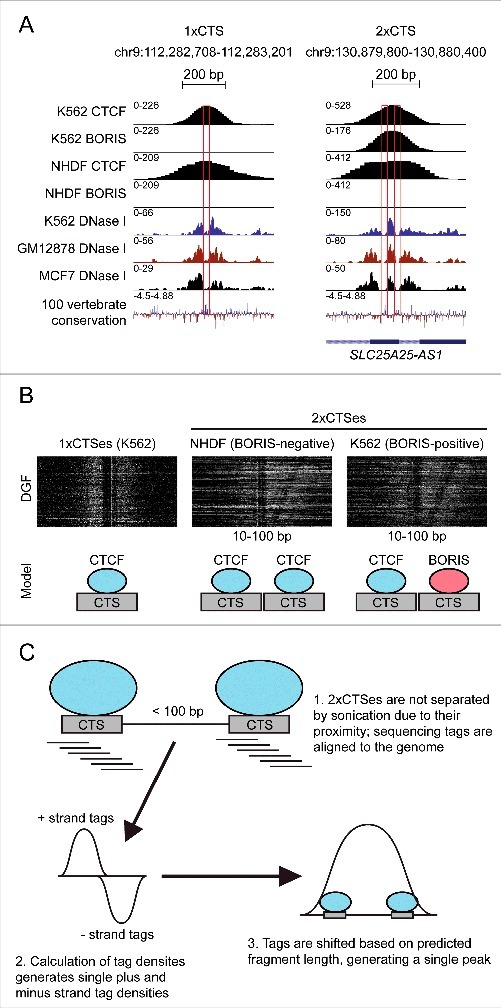
2xCTSes are resolved by nuclease footprinting. (A) UCSC Genome Browser comparison of ChIP-seq and DNase I footprinting data at a selected 1xCTS and 2xCTS regions. K562 CTCF and BORIS ChIP-seq and NHDF BORIS ChIP-seq data are from Pugacheva *et al*.^26^ NHDF CTCF ChIP-seq and DNase I overlap signal data are from the ENCODE consortium. DNase I footprints are boxed in red. (B) Heatmaps showing digital genomic footprinting (DGF) data at 1xCTSes in BORIS-positive K562 cells and 2xCTSes in BORIS-negative NHDF cells and K562 cells. A model of the organization of CTSes and bound molecules within each class of region is shown. (C) Schematic of a possible explanation for the failure of standard ChIP-seq to resolve individual binding sites within 2xCTSes.

To directly elucidate the binding potential of 1x and 2xCTS elements, we performed EMSA analyses using the *in vitro* synthesized central 11 ZF of CTCF. A characteristic double shift was observed with DNA probes derived from 2xCTSes but not 1xCTSes, indicating that two binding events of similar affinity occur within all analyzed 2xCTS-containing DNA sequences. This result also suggests that 2xCTSes are occupied by CTCF homodimers in BORIS-negative cells. Consistent with this, analysis of DNase I footprinting data revealed two closely spaced footprints within 2xCTSes, regardless of whether the cell type analyzed did or did not express BORIS, and single footprints within 1xCTSes. DNase I footprints coincided with regions of conservation, likely representative of conserved consensus motifs. Examples of DNase I footprints and conservation at representative 1xCTS and 2xCTS regions are shown in Fig. 2A, and heatmaps showing aggregated DNase I footprinting data at 1xCTS and 2xCTS regions are shown in Fig. 2B. We conclude that 2xCTSes are indicative of cooperative protein binding events between one molecule of CTCF and one molecule of BORIS or two molecules of BORIS in BORIS-positive cells and two molecules of CTCF in BORIS-negative cells. Such cooperative interactions may be induced by DNA-dependent spatial constraints, perhaps due to molecular crowding-dependent phase separation.^28^

Notably, ChIP-seq analysis of overexpressed BORIS in the BORIS-negative MCF7 cancer cell line recapitulated the specific profile of BORIS binding observed in K562 cells. This result suggests that the primary DNA sequence context within and in the vicinity of 2xCTS-bearing peaks serves as a major determinant of genomic DNA occupancy by CTCF homodimers in normal BORIS-negative somatic cells and also targets replacement of CTCF homodimers BORIS-positive germ or cancer cells with CTCF&BORIS heterodimers and, less often, with BORIS-only homodimers.

Analysis of CTCF and BORIS binding to repetitive elements also revealed striking distinctions between repeat-contained CTCF-only, CTCF&BORIS, and BORIS-only sites.^29^ CTCF-only sites were enriched in evolutionarily ancient and inactive types of repeats, while CTCF&BORIS sites were mainly located in uncharacterized tandem repeats. In contrast, BORIS-only sites were found primarily within the evolutionarily young SVA class of repeats. SVA elements are primate-specific, active retrotransposons^30^ and so their uncontrolled activity presents a threat to the stability of the germline.^31^ Analysis of repeat expression by RNA-seq following BORIS knockdown revealed a widespread upregulation of SVA expression, suggesting that BORIS acts as a repressor of SVA transcription.^29^ Given that SVA repeats are primate-specific, these observations suggest that germline-restricted BORIS continued to evolve after the divergence of the primate lineage, acquiring a specific function in germline defense.

## The regulatory potential of 2xCTS-containing CTCF regions

Aside from their occupancy by BORIS in BORIS-positive cells and the presence of clustered CTCF motifs, 2xCTSes are distinguishable from 1xCTSes by chromatin features and binding of additional proteins. First, 2xCTSes display a histone modification profile consistent with active transcription: they are enriched for H3K4me3, associated with promoter activity, and H3K27ac, linked to active promoters and enhancers. Moreover, 2xCTSes display robust enrichment of the histone variant H2A.Z, which is associated with active transcription, as well as increased chromatin accessibility as measured by DNase I hypersensitivity. Regions containing 2xCTSes were also selectively enriched for transcription factors including ZNF143, C-MYC, and YY1 as well as histone modifying enzymes such as P300 and SET1B.

As in human cancer cells, 2xCTSes in mouse round spermatids are enriched for H3K4me3 and H3K27ac, as well as RNA polymerase II (RNAPII).^26^ Intriguingly, 2xCTSes in round spermatids are also associated with regions that retain histones in mature sperm. We recently extended these observations using ChIP-seq datasets for transcriptional regulators with known roles in male germ cell development and found that, like their counterparts in human cancer cells, mouse round spermatid 2xCTSes are often enriched for transcriptional regulators relative to 1xCTSes.^32^ Consistent with a function in positive transcriptional regulation, genes associated with 2xCTSes bound by transcriptional regulators were expressed more highly than those associated with non-transcriptional regulator-occupied 2xCTSes or 1xCTSes.

Previous studies identified what are now recognized as 2xCTSes in a number of important regulatory regions. For example, two closely-spaced CTCF/BORIS binding sites were found to be required for the activity of the promoter of the *TSP50* gene, encoding a testis-specific protease aberrantly expressed in cancer.^24^ Other studies identified clustered CTCF motifs in alternative *BORIS* promoters,^17^ the promoter of the *NY-ESO-1* gene,^33^ mouse *Igh* enhancers,^34^ the mouse KvDMR1 imprinting control region,^7^ and the *BAX* promoter.^25^

## Genomic 2xCTS sequences and genome architecture

A notable exception to the higher enrichment of transcription factors and chromatin modifiers at 2xCTSes compared to 1xCTSes is the absence of the cohesins RAD21 and SMC3 from BORIS-only regions, suggesting that BORIS alone is insufficient to recruit the cohesin complex. CTCF is well known to cooperate with cohesin in regulating genome architecture,^35^ and increased expression of BORIS in previously BORIS-negative cancer cells could thus rewire genome architecture by replacing one or two CTCF molecules at some of its target regions. Another non-mutually exclusive possibility is that BORIS interacts with an alternative, cancer-testis specific set of architectural factors which are normally co-expressed together with CTCF and BORIS only during gametogenesis. Since BORIS is present in male germ cells during and after meiosis, and so an attractive hypothesis is that BORIS at 2xCTS elements may interact with at least one of three meiosis-specific subunits of cohesin complexes to contribute to a stage-by-stage re-establishment of genome architecture in haploid post-meiotic round spermatids.

Previous work has shown that N-terminal fusion of EGFP to the 11-ZF DNA binding domain of CTCF is sufficient to disrupt the intra-chromosomal loop between the maternal imprinting control region (ICR) and the maternally imprinted *IGF2* gene,^36^ suggesting that the EGFP/11-ZF chimera functions as a dominant-negative decoy that associates with DNA but cannot interact with the appropriate factors to form this loop. As the N- and C- termini of BORIS are highly diverged from those of CTCF, it stands to reason that BORIS might similarly serve as a decoy by both disrupting CTCF-mediated loops and establishing new BORIS-dependent loops.

A specific way in which BORIS overexpression could alter genome conformation is through the disruption of topologically associating domains (TADs). TADs are megabase-scale segments of the genome displaying high levels of self-interaction that are dependent on CTCF for proper folding.^37^ Disruption of TADs is associated with activation of proto-oncogenes in cancers including T-cell acute lymphoblastic leukemia,^38^ glioma,^39^ and colorectal carcinoma.^40^ Thus, even transient overexpression of BORIS, potentially in combination with mutation or loss of genomic CTSes,^41^ dysfunction of CTCF, and/or malfunction of BORIS and/or CTCF-interacting proteins, could be a powerful driving force in carcinogenesis via reorganization of chromatin architecture and concomitant activation of proto-oncogenes.

CTCF is also proposed to mediate the formation of chromatin loops much smaller than TADs.^42^ Such loops are thought to result from pairs of distal CTCF motifs (‘loop anchors') in a convergent orientation. Loss of a CTCF molecule from one loop anchor could thus impair formation of the loop, leading to inappropriate transcriptional consequences.^43^ Notably, our previous work has shown that dual CTCF&BORIS-bound regions are enriched at the RNAPII-bound anchors of chromatin loops specific to K562 cells.^26^ Furthermore, the same regions were occupied by CTCF and RNAPII in BORIS-negative MCF7 cells, but the associated chromatin loops were different.

## Conclusions and future directions

Despite having potentially fundamental implications for understanding the regulatory DNA lexicon responsible for transmission of epigenetic memories by mitotic cancer cells through an aberrantly immortal growth in tumors *in vivo* and in tissue culture *in vitro* and by post-meiotic germ cells in fertile males throughout continuous rounds of normal germ cell development, the majority of distinct single and bipartite 11 ZF binding regions have been overlooked in chromatin studies based on mapping mouse and human CTCF binding sites by ChIP-seq, even in the K562 cell line, which expresses high levels of BORIS and has been a robust model for functional analysis of BORIS.^26,29,44^

Moving forward, it will be of interest to understand the architecture of 2xCTSes and how they might induce protein-protein interactions (PPIs) between molecules of CTCF and/or BORIS. A rather strict limitation on the total length of 2xCTSes (<100 bp between adjacent motifs) suggests that this relatively short spacing may be required for the proximity-induced dimerization shown to occur *in vitro*.^26^ Additionally, a restricted spacer length might be important for exclusion of a single nucleosome between CTCF motifs, which could interfere with DNA-dependent proximity-induced CTCF/BORIS PPIs. To test this, alteration of the spacing between adjacent CTCF motifs in known 2xCTSes followed by EMSA analysis could be employed, followed by *in vivo* editing of 2xCTSes using CRISPR. CRISPR editing of 2xCTSes in cell lines would also be a useful way in which to understand the potential roles of 2xCTSes in the regulation of transcription and chromatin architecture. We also note that CTCF multimerization could be mediated by interactions with RNA, as has been shown for the *Wrap53* antisense transcript of *p53*.^45^ Further study of the structure of 2xCTSes *in vivo* will also require a way to deconvolve closely-spaced binding sites not resolved by standard ChIP-seq. We presume that 2xCTSes are convolved into single peaks because the spacer DNA between CTSes is not sheared during sonication, leading to the accumulation of sequencing tags from two closely-spaced but distinct binding sites into single pileups, leading to assignment of those fragments into single peaks (Fig. 2C). A corollary of this idea is that 2xCTSes would not be resolved by ChIP-exo^46^ or ChIP-nexus,^47^ as CTCF and/or BORIS crosslinked to DNA would act again as a chemically stable exonuclease barrier, preventing digestion of the short intervening stretch of DNA that provides spatial proximity to adjacent CTCF motifs and may contribute to obligatory hetero-dimerization of CTCF and/or BORIS at these regions. However, it is possible that micrococcal nuclease may be able to resolve 2xCTSes on native chromatin, given that it has endo- and exonuclease activities. Resolution of 2xCTSes is unlikely to be achieved by computational methods such as BRACIL^48^ designed for deconvolution of two spatially constrained CTCF binding events, because this and similar mathematical attempts to enhance bipartite binding site resolution would still require an input of short reads that could not be generated by DNA ends absent inside ultra-sonicated but chemically crosslinked 2xCTS-containing chromatin fragments. In contrast, Fig. 2A-B illustrates that DNase I footprinting provides a promising alternative for resolving dual CTCF footprints without fixation of 2xCTS-based chromatin complexes.

It will also be of interest to understand why natural duplication of the highly conserved 11-ZF coding exons of ancestral CTCF gene occurred in spite of potential competition between two 11-ZF DBDs for the same target sequences, as well as to determine how divergence of *BORIS*, the “second CTCF gene” in mammals, would result in strictly testis-specific expression of the human BORIS gene (on chr20q13) so that it could allow ubiquitously-expressed human CTCF (on chr16q22) to avoid functional interference during continuous rounds of human development and reproduction.

Finally, it remains to be determined whether BORIS associates with additional factors involved in the regulation of genome architecture, particularly with meiotic cohesins in normal meiotic and post-meiotic germ cells, and whether these BORIS partners are aberrantly co-activated with BORIS in immortalized cancer stem cells responsible for tumor initiation in BORIS-negative somatic tissues. Co-upregulation of meiotic cohesins with BORIS in cancer could influence genome-wide organization of chromatin and orchestration of transcriptional regulation through the two markedly distinct types of regulatory regions described here. Cataloging of the tens of thousands of CTCF-associated DNA sequences in dozens of human cell lines detected by the ENCODE Consortium (http://www.factorbook.org/human/chipseq/tf/CTCF) will be a valuable step toward understanding “binary CTCF code” emerging in the regulatory DNA language of all heritable epigenomes from combinatorial diversity of two or more individual DNA motifs within CTCF target regions bound equally well by the 11-ZF regions of CTCF and BORIS.
